# Chemical Entity Recognition and Resolution to ChEBI

**DOI:** 10.5402/2012/619427

**Published:** 2012-02-15

**Authors:** Tiago Grego, Catia Pesquita, Hugo P. Bastos, Francisco M. Couto

**Affiliations:** Departamento de Informática, Faculdade de Ciências, Universidade de Lisboa, 1749-016 Lisboa, Portugal

## Abstract

Chemical entities are ubiquitous through the biomedical literature and the development of text-mining systems that can efficiently identify those entities are required. Due to the lack of available corpora and data resources, the community has focused its efforts in the development of gene and protein named entity recognition systems, but with the release of ChEBI and the availability of an annotated corpus, this task can be addressed. We developed a machine-learning-based method for chemical entity recognition and a lexical-similarity-based method for chemical entity resolution and compared them with Whatizit, a popular-dictionary-based method. Our methods outperformed the dictionary-based method in all tasks, yielding an improvement in *F*-measure of 20% for the entity recognition task, 2–5% for the entity-resolution task, and 15% for combined entity recognition and resolution tasks.

## 1. Background

Biomedical literature provides extensive information that is not covered in other knowledge resources and the amount of information produced and published in articles and patents is growing at a fast pace, thus the manual analysis and annotation of the literature is a tedious, time-consuming, and costly process. Fortunately, this process has been addressed by text-mining systems that have already shown to be helpful in speeding up some steps of this process [1]. Normally, the first step of text-mining systems is the identification of named entities in text. This is a crucial step and includes the tasks of named entity recognition and entity resolution. Named entity recognition comprises the identification of the text boundaries that limits a string referring to a target category, such as chemicals [2]. Entity resolution takes as input the strings identified in the previous task, in order to find exactly which chemical each string corresponds to, by mapping each of them to a reference database entry.

Most efforts in entity recognition and resolution have been made in the identification of protein and gene named entities in the literature. The performance of systems tackling such tasks has been measured in competitions such as the BioCreative challenge [3, 4], TREC Genomics Track [5] and the NLPBA challenge [6]. However, few efforts have been made on the recognition and resolution of other terminologies, partly due to the lack of annotated corpora and the high costs associated to its generation. One of such cases is chemical terminologies, a field that suffers from the lack of available corpora but can benefit immensely from text mining. For example, chemical metabolites are essential for proteomics and transcriptional network studies, areas that benefit greatly by text-mining systems [7, 8].

There are two main specific challenges in chemical entity recognition: the first is the potential infinite number of compounds since new chemicals are constantly being synthesized; the second is the high ambiguity in chemical representation, with a single chemical being described in publications by trivial names, systematic names, registry numbers or formal descriptions like formulas. Therefore, the creation and maintenance of chemical terminologies is a complex task [9].

Named entity recognition systems employ two main approaches [10].

- Dictionary-based approaches require domain terminologies to find matching entities in the text. This approach, however, depends on the availability and completeness of these terminologies and is limited to the entities contained in them and given the vast amount of possible chemical compounds, the terminologies are always incomplete. On the other hand, terminologies are also ambiguous, since some terms are common English words which will produce false positives. An advantage of this approach is that entity resolution is directly obtained by the name entity recognition task, since each entity recognized is inherently linked to an individual term of the terminology. Advanced techniques used by these approaches include enhancement of the input terminologies by integrating and normalizing different databases, improving string-matching methods more suited for the target class of entities, and the development of rules for postprocessing to refine the results.
- Machine-learning-based approaches require an annotated corpus which is used to learn a model that can be applied for named entity recognition in new text. Systems using this approach use named entity recognition as a classification task that tries to predict if a set of words represent an entity or not. The bottleneck of this approach is the availability of an annotated corpus large enough to enable the creation of an accurate classification model, and the need for an entity-resolution module for mapping the recognized entities to database entries.

Earlier efforts in chemical entity recognition include the comparison between a dictionary-based approach and a machine-learning-based approach. Namely, a study compared a dictionary-based segmentation method with Naïve Bayesian classification methods for recognizing chemical names using the Unified Medical Language System (UMLS) Methasaurus for training and testing [11]. The Naïve Bayesian method obtained the highest result, 97% accuracy, but it was trained and tested in a single lexicon terminology (UMLS) and biomedical literature contains a much less standardized chemical nomenclature. A dictionary-based system using a set of rules that rely upon lexical and dictionary information was reported having 90% *F*-measure in identifying chemical compounds [12], but evaluation was performed in only 55 abstracts selected by acetylation-related keywords which does not provide a sound evidence for how extensible these results are in a larger and broader corpora. Wren [13] developed a first-order Markov Model to distinguish chemical names from words using the ChemIDplus database [14] as positive training and reports an average precision of 83% in extracting chemical terms from MEDLINE abstracts. Klinger et al. [15] presents a machine-learning approach based on conditional random fields (CRF-), and a performance of 80–85% *F*-score. However, this system is for detection of systematic (IUPAC-like) chemical names, where it is usual for a chemical to be referenced by the trivial name or other synonyms. Oscar3 is an open-source system that uses an extensible internal lexicon and several natural language-processing methods for the automated annotation of chemicals in biomedical journal articles, reporting an *F*-score of 80% [16]. Whatizit is a popular text-processing system capable of identifying a wide variety of biomedical terms, including chemicals, by using several pipelines, each one based on a terminology [17]. One of the available pipelines is based on Chemical Entities of Biological Interest (ChEBI).

ChEBI is a freely available dictionary of molecular entities, containing also groups (parts of molecular entities) and classes of entities that enable ChEBI to be organized as a chemical ontology, structuring molecular entities into subsumption classes, and defining the relations between them [18]. It is not as comprehensive as other dictionaries but is manually curated which guarantees high quality. The ultimate goal of ChEBI is to provide and promote a gold standard for annotation of molecular entities, which comprises a controlled vocabulary (standardized and unambiguous terminology), graphical representations of molecular structure (clear and unambiguous 2D diagrams), and defined logical relationships between concepts (ontology). Each entry of ChEBI is identified by a unique identifier, a name, and when appropriate a definition and synonyms.

Despite the lack of available annotated corpora to promote and evaluate chemical entity identification systems, a joint team of curators from ChEBI and the European Patent Office has manually annotated chemical named entities in a set of 40 patent documents. This annotated corpus was released http://chebi.cvs.sourceforge.net/viewvc/chebi/chapati/patentsGoldStandard/ in 2009 as a gold standard to aid the development of text-mining tools.

Due to the release of a gold standard of patent documents and the increasing availability of high-quality chemical terminologies such as ChEBI, we decided to develop a novel chemical entity identification system to assess how the availability of this new domain knowledge can enhance the performance of text-mining systems. Thus, the contributions of this paper are the following:

- the development of a chemical named entity recognition method based on a machine learning approach;
- an entity-resolution method that maps recognized entities to the ChEBI database based on lexicon similarity approach;
- the enrichment of mapped entities in the gold standard of patent documents;
- a performance assessment of our system in comparison to the results obtained by the popular text mining system, Whatizit.

## 2. Results

In this section, we will present an assessment of our machine-learning-based method in comparison to the dictionary-based method, Whatizit. Both methods were applied to the gold standard, but only 47% of the total amount of named entities of this corpus were mapped to ChEBI at that time by the curators. However, since that time, the size of the ChEBI dictionary has almost doubled in the number of compounds. Thus, we decided that an enrichment in the mapping of the annotated entities was necessary to significantly improve the amount of chemical named entities mapped to ChEBI.

Both tasks of chemical entity identification process have been evaluated using the enriched gold standard. In the first task, named entity recognition, we evaluated the ability of each method to recognize chemical named entities in text. In the second task, chemical entity resolution, we assess the ability of each method to map the recognized chemical entities to the ChEBI database, that is, associating a correct and unique ChEBI identifier to each named entity recognized.

### 2.1. Gold Standard

The gold standard of patent documents was developed to promote the enhancement of text-mining tools for identifying chemicals not only in patents but also within all biomedical literature.

This manually annotated corpus contains a total of 18,061 chemical entities recognized in its 4,985 sentences. When possible, the curators included a mapping from the recognized chemical entities to a ChEBI identifier, achieving a total of 8,528 mappings to ChEBI identifiers, that is, 47.2% of the total amount of chemical entities. The relatively low amount of mappings is not only due to the novelty inherent to patent documents, but also because ChEBI is still a recent project under rapid growth.

**Figure 1:**
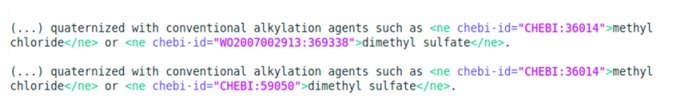
Example of mapping enrichment in the corpus. The first line shows the original entities corpus, where one entity was not mapped to ChEBI. On the second line, we show the corpus after enrichment, where that entity could be mapped.

To increase the amount of available mappings, we checked all previously unmapped entities in the gold standard for a valid ChEBI identifier in an up-to-date release of ChEBI. We were able to increase by 13.7% the amount of mappings to ChEBI, that is, to 9,696 entities, 53.7% of the total entities in the gold standard.  Figure 1 shows an example of a sentence containing two chemical entities, from which only one was originally mapped to ChEBI (methyl chloride). After our enrichment process, the second entity (dimethyl sulphate) was also mapped. Our enriched version of the gold standard is also publicly available on demand.

### 2.2. Named Entity Recognition

Typically, the evaluation of named entity recognition considers exact matching (correct matching of both the left and the right boundary of the named entity) as the most precise assessment. However, exact matching is very strict and sometimes a relaxed assessment, such as partial matching, can produce more useful results [19]. Partial matching evaluation is the most relevant for tools targeted at semisupervised tasks, such as aiding curators in finding target entities through literature analysis. In these cases, a partial identification is sufficient to successfully highlight the presence of entities for manual validation. In partial matching, correct recognition is assumed when any fragment of the named entity is correctly identified in the text. We assessed the results against exact matching criteria, and also against relaxed matching criteria such as the left matching, right matching, left/right matching, and partial matching. The results were obtained for the two chemical entity recognition methods: the dictionary-based method using the Whatizit tool and our machine-learning-based method (see Section 5 for their description).  Table 1 shows these results in the chemical entity recognition task.

For all assessments, our machine-learning method performed better than the dictionary-based method (scoring *F*-measures on average *~*20% than those obtained with the dictionary-based method). The dictionary-based method recognized a similar number of entities to the ones present in the gold standard (18,683). The machine learning method recognized a lower amount of entities (13,832), however, with a much higher precision and recall. Assessments against the right boundary consistently yielded slightly better results than the assessments against the left boundary, for both methods. For the exact matching evaluation, the dictionary-based method obtained an *F*-measure of 32%, while our machine-learning method obtained 57%, that is, having both much higher precision and recall. For a partial matching, a top *F*-measure of 77% was achieved by our machine-learning method, while the dictionary-based method achieved 70%. Both methods had a similar recall and the difference was made by the higher precision of the machine-learning method (20% higher precision).

The difference in the performance between the two methods might be explained by the fact that several entities in the annotated corpus do not have a valid ChEBI identifier (i.e., they do not yet exist in the ChEBI dictionary). That makes it impossible for a ChEBI dictionary-based method to find those entities that account for almost 50% of the total entities in the annotated corpus. The machine-learning method does not have this bottleneck and is suited to identify novel compounds not yet present in the database, and thus to aid in database extension. However, to avoid this bias, we analyzed the entity recognition performance only considering the entities in ChEBI, that is, using only the subset of entities manually mapped to ChEBI in the annotated corpus (9,696 out of the 18,061).  Table 2 shows these results obtained for both methods.

We can check that now the amount of entities recognized and mapped by the machine-learning method (using the entity resolution method described in Section 5) is similar to the number of entities mapped to ChEBI in the corpus. Even under these conditions, the machine-learning method continues to perform better than the dictionary-based method, on average an *F*-measure *~*15% higher. However, the amount of true positives is very similar for both methods and the decisive factor is the higher amount of false positives (low precision) generated by the dictionary-based method.

### 2.3. Entity Resolution

In this task, we aim at mapping the recognized chemical entities to the appropriate term in the ChEBI database. The evaluation consists in comparing the mappings produced by both automatic methods with the manual mappings in the gold standard. This means that a true positive is not only a chemical entity that has been correctly recognized, but also correctly mapped to a ChEBI identifier. Thus, in order to measure the difference in performance between the two methods, both were tested against the subset of entities manually mapped to ChEBI in the corpus.  Table 3 shows the results achieved by both methods in correctly recognizing and mapping chemical entities to ChEBI.

We can see that the *F*-measure yielded by our methods is consistently higher than the one of the dictionary-based method by 12–14%. These results are dependent of the entity recognition results, shown in Table 2, since a true positive in Table 3 means that it must be also a true positive in Table 2. Thus, a new assessment was made to evaluate only the entity-resolution task. It consists in restricting the resolution task to the chemical entities that have been correctly recognized simultaneously by both methods. The number of entities recognized is the intersection of the entities correctly recognized by both methods.  Table 4 shows the results from this assessment.

We see that entity-resolution results are similar for both methods, with a slight advantage for our entity-resolution method described in Section 5.

## 3. Discussion

### 3.1. Named Entity Recognition

Table 1 shows that our machine-learning method outperforms the dictionary-based method at all evaluations and assessments, with the exception of the recall for a partial matching assessment where the dictionary-based method obtains a slightly better result with *~*2% better recall, but at the cost of 11% decrease in precision. However, there are entities annotated in the gold standard for which no ChEBI identifier could be given by curators even after enrichment, which indicates its absence from the dictionary and the impossibility of the dictionary-based approach to find those entities, thus lowering the recall of the dictionary-based method.

This issue was addressed by the evaluation whose results are shown in Table 2, where only the subset of entities in the gold standard that are mapped to ChEBI were used. The recall of the dictionary-based approach does increase to values similar to those of the machine-learning approach, but precision remains much lower which is a drawback for *F*-measure. Independently of the type of assessment, both systems are able to identify about 60–70% of the chemical entities, but precision is consistently higher for the machine-learning method (15–25% higher).

To understand this difference in precision, we analyzed the most common recognition errors of both systems, and found that some of the most frequent entity recognition errors of the dictionary-based method include terms such as *can*, *group,* and *all*, which are common English words widely used in a nonchemical context and accounted as false positives. In fact, those terms are listed in ChEBI as synonyms of *calcium(0) (CHEBI: 29320)*, *group (CHEBI: 24433),* and *allose (CHEBI: 37690),* respectively, but are never used in the corpus in a chemical context. These entity recognition errors contribute to the low precision of the dictionary-based method.

A systematic annotation error of the machine-learning method is the annotation of *R* as a chemical entity. Although this term is used in the corpus to represent generic chemical groups, it was not considered by the curators as a chemical entity. Frequent annotation errors of both approaches include terms that are in fact chemical entities, such as *serine*, *drug,* and *water*. However, in the case of *serine,* it is used frequently in one of the documents in the context of a protein (*serine protease*) and thus curators decided not to consider it a chemical entity during manual annotation. The other two terms were also not considered by the curators given their low information content, which might lead to under-annotation in the corpus.

When looking at the assessment against the right boundary alignment, several chemical groups have been partially identified. In the case of the dictionary-based method, for instance, the terms *acid*, *amine,* and *ester* have been frequently recognized but the correct left boundary was not. The machine-learning approach deals much better with these examples and can usually identify both boundaries of *acid* terminating entities. However, the machine-learning approach frequently makes this mistake with other terms such as *alkyl* and *aryl*. The right boundary is correctly identified while the left one is not, mostly due to the complexity of the annotated term (e.g., *substituted or nonsubstituted lower alkyl*). These terms are not identified by the dictionary-based method, because only *alkyl group* and *aryl group* are terms in ChEBI. For the left boundary assessment, no systematic errors have been identified.

### 3.2. Entity Resolution

The difference between the true positives of Tables 2 and 3 shows that for the exact matching assessment 80% of the entities recognized by the dictionary-based method have been correctly mapped to ChEBI, and 82% using our methods. This shows that our methods perform slightly better than the dictionary-based method in the entity identification process.

We analyzed the resolution of entities that failed by the dictionary-based method and found that the mapping for these three terms *trehalose*, *nicotine,* and *mannitol* were the most frequent errors. These terms were manually annotated with the ids CHEBI: 27082, CHEBI: 18723; CHEBI: 29864, respectively, and their name is the term itself. However, those terms have been annotated by the dictionary-based method as *α*, *α-trehalose (CHEBI: 16551)*, *(S)-nicotine (CHEBI: 17688),* and *D-mannitol (CHEBI: 16899),* respectively. This happened because those terms contain the original term listed as a synonym. In these cases, the curators tended to selected the more generic term while the dictionary-based method the most specific and common form of the molecule.

In the case of the machine-learning method, those terms were correctly mapped, but at the top of the most frequent errors, we find the terms CN and OH. These terms were manually annotated as *cyano group (CHEBI: 48819)* and *hydroxy group (CHEBI: 43176)*, but erroneously mapped as *ununbium atom (CHEBI: 33517)* and *ethanol (CHEBI: 16236)*. Neither of the entries in ChEBI contain the original term listed as a synonym but instead have listed –CN and –OH. Those terms (with the hyphen) should have been used instead, so they could properly represent the entities as chemical groups. The used terms (without the hyphen) make it hard to correctly map the entities to ChEBI. The dictionary-based method does not even recognize these terms as chemical entities.

In Table 4, a comparison was made about the efficiency of the entity resolution of both methods, because only named entities correctly recognized by both approaches are considered and the focus of evaluation is the performance of the mapping of those named entities. Precision of the resolution is around 80% higher with an exact matching assessment and decreasing for more relaxed assessments. The precision of our entity-resolution method is consistently higher (3–7%) than the precision of the dictionary-based method.

## 4. Conclusions

Our work started by enriching the mapping of chemical entities to ChEBI in a manually annotated gold standard corpus of patent documents. This enabled a proper evaluation of entity-resolution tasks in addition to entity recognition tasks. Using this corpus, we developed a machine-learning method for chemical named entity recognition and we compared its performance against the popular dictionary-based method, Whatizit.

Results show that the dictionary-based method can already provide competitive results in recognizing chemical named entities, obtaining an *F*-measure of up to 70%. However, our machine-learning method outperformed the dictionary-based method, by having an *~*20% average increase in the *F*-measure. A known drawback of dictionary-based methods is the inability to recognize entities not present in the dictionary used, and many such entities were present in the corpus. So we tested both systems using only the subset of entities in the corpus to which curators had assigned a ChEBI identifier. Still, the machine-learning method outperformed the dictionary-based method with a *~*15% average increase in the *F*-measure.

The dictionary-based method intrinsically performs the resolution of the recognized entities, but for the machine-learning method a resolution method is required. Thus, we developed a resolution method based on lexical similarity for mapping chemical entities to ChEBI, which was used to perform the resolution of the recognized chemical entities by our machine-learning approach. This method has shown to be effective, surpassing the dictionary-based method in entity-resolution task by 2–5% in *F*-measure.

Analyzing the process of entity identification (combined recognition and resolution tasks), the machine-learning method combined with lexical similarity outperformed the dictionary-based method by an average of *~*15% *F*-measure.

Overall, we demonstrated that a completely dictionary independent machine learning entity recognition method and a lexical similarity resolution method can surpass dictionary-based methods in recognizing chemical compounds and mapping them to the ChEBI database. In addition, our method has the ability to find novel entities and aid in the extension of chemical data resources. The top result of 77% *F*-measure is a promising result and makes this system useful in semisupervised tasks.

Future work will focus on automatically reducing the number of recognition errors, by taking advantage of the ontology structure of ChEBI. To this end, we will explore the fact that a given document has a limited focus, and thus will contain chemical entities which are somehow related to each other. Using semantic similarity methods over the ChEBI ontology, we expect to measure the confidence on an automatic entity identification by its semantic similarity with other entities also identified in the text [20].

## 5. Methods

### 5.1. Dictionary-Based Method

 Dictionary-based methods normally create lists containing synonyms and term variants from existing data resources and match them to the literature. This approach relies on the availability and completeness of terminologies to find matching entities in the text. Whatizit is a popular text processing system that performs exact matching between the terms in a lexicon and the terms in the literature. Several lexicons are available in Whatizit to use through pipelines that annotate most kinds of biomedical entities. ChEBI is one of the dictionaries available in Whatizit, through the pipeline whatizitChebiDict.

The pipeline whatizitChebiDict can be accessed by web services, taking as input an XML file which consists of the text we want to process between text tags. As output Whatizit provides the XML file with the recognized entities between ne tags, the entity resolution is given by the corresponding ChEBI identifiers assigned as attributes to the tag.

**Figure 2:**
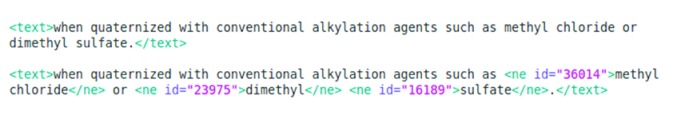
Example of Whatizit. The first line shows a small example of an input to whatizit. The second line shows the output, where the identified entities were marked and mapped to ChEBI identifiers.

Figure 2 shows an example of a piece of text given to Whatizit through the whatizitChebiDict and the corresponding result of entity identification.

### 5.2. Machine-Learning Method

Conditional random fields (CRFs) [21] have been very successfully used in biomedical named entity recognition tasks [22], so we decided to use the CRF implementation of MAchine Learning for LanguagE Toolkit (MALLET) [23] for the development of our machine-learning method. MALLET is a Java-based package for statistical natural language processing, document classification, clustering, topic modeling, information extraction, and other machine-learning applications to text.

The first step in the entity recognition process is the splitting of the text into a sequence of tokens. The tokenization was performed using a specifically adapted tokenizer for chemical text proposed by [24].

A set of tags was defined in order to properly denote the boundaries of the named entities present in the corpus. The set of tags is composed of five tags, namely, NO (nonchemical token), NE (single-token chemical entity), S-NE (start token of a multi-token chemical entity), M-NE (middle token of a multitoken chemical entity), and E-NE (end token of a multitoken chemical entity). For example, the sentence *“…an oligomeric amdioamine salt and an amidoquat…”* would be correctly tagged by the sequence of tags *NO, S-NE, M-NE, E-NE, NO, NO, NE*.

Each token is represented as a feature set (plus the correct tag in the training), which includes the stem of the token, prefix and suffix of the token and indication if the token is a number.

For example, for the excerpt “…cosmetic compositions containing colostrum, *tocopherols*, *zinc oxide* and *hyaluronic acid‌*…” (the chemical entities present are in italic) the list of tokens obtained by the tokenizer and the feature sets are shown in Table 5.

A richer feature set could be used, especially with the aid of chemical data resources that can provide, for instance, the information if the token is included in the dictionary, and other data such as frequencies for chemical suffixes and prefixes. The efficiency of the case-based method can certainly be improved using this richer sets of features that take advantage of the knowledge provided by such chemical resources, but for this study, we were interested in a fully dictionary independent approach to be compared with a fully-dictionary-based approach, so no dictionary-based features were used.

The CRF implementation uses a sequence of sets of such features, plus a label (for the documents in the training set) for the training step. The resulting model can then be used to predict the label of another sequence of features (the testing set).

In the chemical entity recognition performed by our machine learning approach, each one of the 40 documents was annotated using a model generated using the remaining 39 documents as a training data, using a leave-one-out crossvalidation approach.

The output of this method contains the chemical named entities that could be identified but does not map those entities to the ChEBI dictionary. An entity-resolution method is required to perform the mapping of the identified entities.

### 5.3. Entity-Resolution Method

The entity-resolution method we used is an adaptation of our lexical-similarity method used in the ontology matching algorithm BLOOMS [25] which in turn is based on FiGO, a methodology for finding GO terms in text [26]. It takes as input the string identified as containing a chemical compound name and returns the ChEBI identifier it corresponds to along with a confidence score. The method is composed of two sequential approaches: exact match and partial match.

In the exact match, we determine if the input string contains any descriptors of ChEBI terms, that is, their names and synonyms. If an exact match is found, the corresponding ChEBI identifier is returned along with a confidence score. Here, we used a confidence score of 1, when the match is to the name of a term, and of 0.8 when the match is to a synonym.

If no exact match is found, the partial match method is run. It relies on shared words between the input string and the ontology terms names and synonyms. In a preprocessing step, the ontology vocabulary (the set of all textual information contained in an ontology in the form of names and synonyms) is processed through tokenization and removal of stopwords, to generate the list of ontology words. Then, the evidence content of each ontology word is calculated as the negative logarithm of the relative frequency of a word in the ontology vocabulary:

(1) $$\begin{array}{c} EC\left(w\right)=-\log f\left(w\right), \end{array}$$

where *f*(*w*) is the frequency of the word in the vocabulary of an ontology.

The final frequency of a word corresponds to the number of terms that contain it in their descriptors (names or synonyms). This means that a word that appears multiple times in the descriptors of a term is only counted once, preventing bias towards terms that have many synonyms with similar word sets. The evidence content of ontology words and the presence of words in ontology terms are stored in a database to support the partial match algorithm.

When the partial match algorithm is run, the input string is processed in a similar fashion, via tokenization and stopword removal. Then, ontology descriptors that share words with the input string are retrieved as partial matches. The final score, Sim_PM_, for each partial match between the input string and a term descriptor is given by a Jaccard similarity, which is calculated by the number of words shared by the two concepts, over the number of words they both have, with each word being weighted by its evidence content:

(2) $$\begin{array}{c} \text{Si}{\text{m}}_{\text{PM}}=\text{desc}\times \frac{\sum_{w\in (\text{input}\cap t_{d})}EC\left(w\right)}{\sum_{w\in (t_{d})}EC\left(w\right)}, \end{array}$$

where desc is a weighting factor corresponding to whether the match is made to a term's name or synonym, *w* are the ontology words contained in the input string and descriptor of the term (*t*_*d*_), and EC is the evidence content of a word. Here, we used a desc value of 1 for matches to names and of 0.8 for matches to synonyms. Sim_PM_ provides a measure of the relevance of the words shared by the input string and the term descriptor versus the total relevance of words in the term's descriptor. The partial matches are ranked by this score, and the method returns the ChEBI identifier corresponding to the descriptor with the highest score, for each input entity.

## Figures and Tables

**Table 1:** Evaluation of entity recognition, full gold standard of 18,061 chemical entities. Results of named entity recognition for each assessment and method are shown in this table. The dictionary method recognized a total of 18,683 entities while the machine-learning method recognized 13,832 entities. True positives (TP) is the amount of entity recognitions that agree with the gold standard for each assessment. Values of precision, recall, and *F*-measure are presented.

Assessment	Method	TP	Precision	Recall	*F*-measure
Exact matching	Dictionary	5,868	31.41	32.49	31.94
	Machine learning	9,094	65.76	50.35	**57.03**
Left matching	Dictionary	6,868	36.76	38.03	37.38
	Machine learning	9,892	71.53	54.77	**62.04**
Right matching	Dictionary	8,015	42.90	44.38	43.63
	Machine learning	10,419	75.34	57.69	**65.34**
Left/right matching	Dictionary	9,015	48.25	49.91	49.07
	Machine learning	11,217	81.11	62.11	**70.35**
Partial matching	Dictionary	12,780	68.40	70.76	69.56
	Machine learning	12,328	89.15	68.26	**77.32**

**Table 2:** Evaluation of entity recognition, subset of the gold standard composed by 9,696 chemical entities that contain a mapping to ChEBI. Results of entity identification (named entity recognition and resolution) for each alignment and method are shown in this table. The dictionary method recognized and mapped a total of 18,683 entities while the machine-learning method recognized and mapped 10,681 entities. True positives (TP) is the amount of entity recognitions that agree with the gold standard for each assessment. Values of precision, recall, and *F*-measure are presented.

Assessment	Method	TP	Precision	Recall	*F*-measure
Exact matching	Dictionary	5,651	30.25	58.28	38.83
	Machine learning	5,830	54.60	60.13	**57.23**
Left matching	Dictionary	5,913	31.65	60.98	41.67
	Machine learning	6,084	56.98	62.75	**59.72**
Right matching	Dictionary	6,158	32.96	63.51	43.40
	Machine learning	5,948	55.70	61.34	**58.39**
Left/right matching	Dictionary	6,435	34.44	66.37	45.35
	Machine learning	6,307	59.07	65.05	**61.91**
Partial matching	Dictionary	7,654	40.97	78.94	53.94
	Machine learning	6,703	62.78	69.13	**65.80**

**Table 3:** Evaluation of entity identification, subset of the gold standard composed by 9,696 chemical entities that contain a mapping to ChEBI. Results of entity identification (named entity recognition and resolution) for each alignment and method are shown in this table. The dictionary method recognized and mapped a total of 18,683 entities while the machine-learning method recognized and mapped 10,681 entities. True positives (TP) is the amount of entity recognitions that agree with the gold standard and for which the mapping also agrees with the gold standard. Values of precision, recall, and *F*-measure are presented.

Assessment	Method	TP	Precision	Recall	*F*-measure
Exact matching	Dictionary	4,530	24.25	46.72	31.93
	Machine learning	4,783	44.79	49.33	**46.95**
Left matching	Dictionary	4,559	24.40	47.02	35.13
	Machine learning	4,972	46.56	51.28	**48.81**
Right matching	Dictionary	4,592	24.58	47.36	32.36
	Machine learning	4,885	45.75	50.38	**47.95**
Left/right matching	Dictionary	4,621	24.73	47.67	32.57
	Machine learning	5,074	47.52	52.33	**49.81**
Partial matching	Dictionary	5,185	27.75	53.48	36.54
	Machine learning	5,202	48.72	53.65	**51.07**

**Table 4:** Evaluation of entity resolution, subset of the gold standard composed by 9,696 chemical entities that contain a mapping to ChEBI. Results of entity resolution for each assessment and method are shown in this table. Have been considered for this evaluation only the entities successfully recognized by both methods. For an exact matching assessment, the amount of entities successfully recognized by both methods was 3,668. For the left, right, left/right, and partial matching assessments, that amount was correspondingly 4,022, 4,082, 4,455, and 5,286 entities. True Positives (TP) is the amount of those entities for which the resolution was correct, that is, the mapping agrees with the gold standard. Values of precision, recall, and *F*-measure are presented.

Assessment	Method	TP	Precision	Recall	*F*-measure
Exact matching	Dictionary	3,079	83.94	31.76	46.08
	Machine learning	3,206	87.40	33.07	**47.98**
Left matching	Dictionary	3,215	79.94	33.16	46.87
	Machine learning	3,381	84.06	34.87	**49.29**
Right matching	Dictionary	3,191	78.17	32.91	46.32
	Machine learning	3,467	84.93	35.76	**50.33**
Left/right matching	Dictionary	3,327	74.68	34.31	47.02
	Machine learning	3,650	81.93	37.64	**51.59**
Partial matching	Dictionary	3,861	73.04	39.82	51.54
	Machine learning	4,273	80.84	44.07	**57.04**

**Table 5:** Example of a sequence of features, and the corresponding label (Tag).

Token	Stem	Prefix	Suffix	Number	Tag
cosmetic	cosmet	cos	tic	No	NO
compositions	composit	com	ons	No	NO
containing	contain	con	ing	No	NO
colostrum	colostrum	col	rum	No	NO
tocopherols	tocopherol	toc	ols	No	NE
zinc	zinc	zin	inc	No	S-NE
oxide	oxid	oxi	ide	No	E-NE
and	and	and	and	No	NO
hyaluronic	hyaluron	hya	nic	No	S-NE
acid	acid	aci	cid	No	E-NE
